# Congenital intestinal malrotation presenting as ileal volvulus in an adult woman: a case report and literature review

**DOI:** 10.1093/jscr/rjag249

**Published:** 2026-04-09

**Authors:** Roccantonio Pellegrino, Maria Bonaddio, Marco Giurdanella, Giuseppe Bonadio, Gianlorenzo Schicchi, Vittorio Tedesco, Carmine Gabriele, Luigi Strangis, Francesca Ibba, Denise Gambardella, Ettore Caruso, Manfredo Tedesco

**Affiliations:** Department of General Surgery, “Giovanni Paolo II” Hospital, Via Senatore Arturo Perugini, 88046, Lamezia Terme (CZ), Italy; Department of General Surgery, “Giovanni Paolo II” Hospital, Via Senatore Arturo Perugini, 88046, Lamezia Terme (CZ), Italy; Department of Radiology, “Giovanni Paolo II” Hospital, Via Senatore Arturo Perugini, 88046, Lamezia Terme (CZ), Italy; Department of Anesthesiology, “Giovanni Paolo II” Hospital, Via Senatore Arturo Perugini, 88046, Lamezia Terme (CZ), Italy; Department of Medical and Surgical Sciences, “Magna Graecia” University of Catanzaro, Viale Europa, località Germaneto, 88100 Catanzaro (CZ), Italy; Department of Medical and Surgical Sciences, University of Plovdiv, 15A Vasil Aprilov Blvd, 4002 Plovdiv, Bulgaria; Department of General Surgery, “Giovanni Paolo II” Hospital, Via Senatore Arturo Perugini, 88046, Lamezia Terme (CZ), Italy; Department of General Surgery, “Giovanni Paolo II” Hospital, Via Senatore Arturo Perugini, 88046, Lamezia Terme (CZ), Italy; Department of General Surgery, “Giovanni Paolo II” Hospital, Via Senatore Arturo Perugini, 88046, Lamezia Terme (CZ), Italy; Department of General Surgery, “Giovanni Paolo II” Hospital, Via Senatore Arturo Perugini, 88046, Lamezia Terme (CZ), Italy; Department of General Surgery, “Giovanni Paolo II” Hospital, Via Senatore Arturo Perugini, 88046, Lamezia Terme (CZ), Italy; Department of General Surgery, “Giovanni Paolo II” Hospital, Via Senatore Arturo Perugini, 88046, Lamezia Terme (CZ), Italy

**Keywords:** congenital intestinal malrotation, ileal volvulus, intestinal obstruction, emergency surgery, computed tomography, adult presentation

## Abstract

Congenital intestinal malrotation is a rare developmental anomaly that typically presents during childhood but may remain undiagnosed until adulthood, when symptoms can arise abruptly due to volvulus or intestinal ischemia. We report the case of a 33-year-old woman, previously healthy, who presented with acute abdominal pain, abdominal distension, and peritoneal signs. Laboratory tests revealed mild neutrophilic leukocytosis. Abdominal computed tomography with oral Gastrografin demonstrated marked gastric distension and mesenteric swirling, initially suggestive of intestinal volvulus. Urgent surgical exploration revealed congenital intestinal malrotation with abnormal positioning of bowel loops and a volvulated ileal segment showing vascular congestion and early ischemic changes. Derotation and careful assessment of bowel viability were performed; due to persistent ischemic changes, resection of the compromised ileal segment with primary mechanical anastomosis was required. Postoperative recovery was uneventful. This case highlights the diagnostic challenges of intestinal malrotation in adults, where clinical presentation is often nonspecific and radiologic findings may be misleading. Early surgical intervention remains essential to prevent irreversible ischemic injury and bowel necrosis.

## Introduction

Congenital intestinal malrotation results from incomplete rotation and fixation of the midgut during embryogenesis. While it is usually diagnosed during infancy, a small proportion of cases remain clinically silent until adulthood, when complications such as volvulus may lead to acute and potentially life-threatening presentations [1].

Variants of malrotation (non-rotation, incomplete rotation, and malfixation) predispose patients to midgut torsion due to a narrow and unstable mesenteric root [2, 3]. In adults, diagnosis is frequently delayed because symptoms are often intermittent or mimic more common gastrointestinal disorders [4].

We report a case of symptomatic congenital intestinal malrotation presenting with acute ileal volvulus in an adult woman and emphasize the importance of early surgical management.

## Case description

A 33-year-old woman with no significant medical history presented with acute abdominal pain, abdominal distension, nausea, and absence of bowel movements for three days, while passage of flatus was preserved. Physical examination revealed signs of peritoneal irritation.

**Figure 1:**
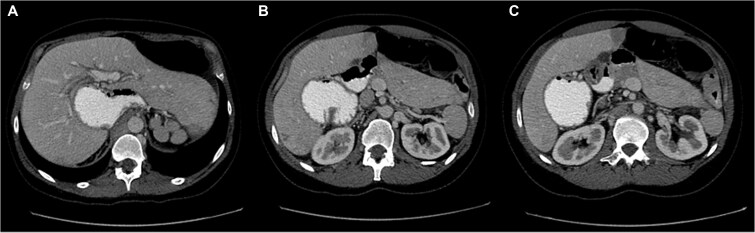
Sequential axial CT images following oral Gastrografin administration showing marked gastric distension and mesenteric vascular swirling, suggestive of volvulus in the setting of intestinal malrotation (A–C).

Laboratory investigations showed mild neutrophilic leukocytosis. Abdominal computed tomography (CT) with oral Gastrografin demonstrated marked gastric distension and mesenteric vascular swirling, suggestive of intestinal volvulus and raising suspicion of an underlying rotational anomaly (Fig. 1).

**Figure 2:**
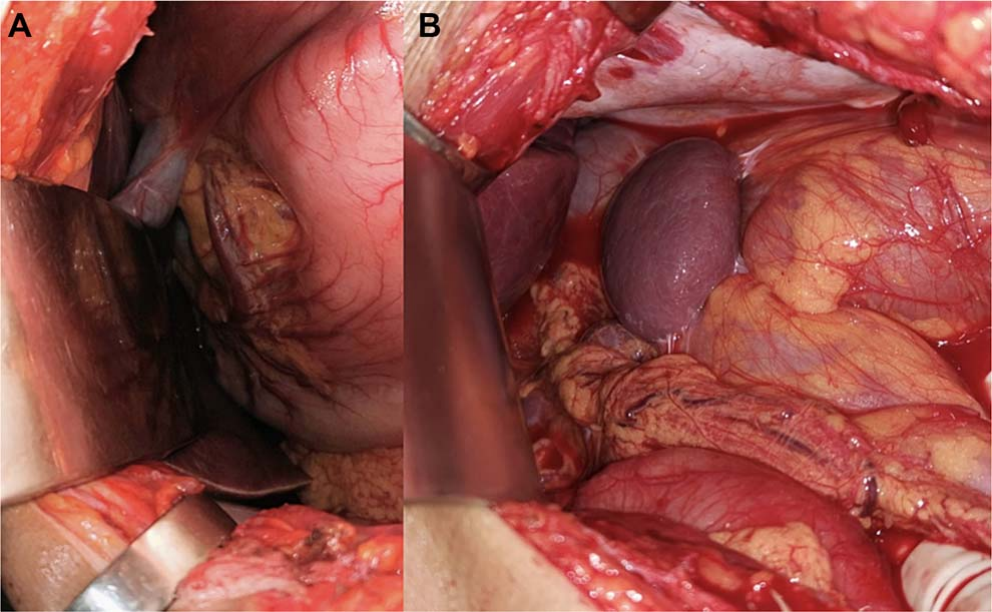
Intraoperative findings during exploratory laparotomy demonstrating: (A) abnormal positioning of intestinal loops consistent with congenital malrotation. (B) Congested ileal segment with early ischemic changes secondary to volvulus.

Urgent exploratory laparotomy was performed. Intraoperatively, congenital intestinal malrotation was confirmed, with rightward deviation of the gastric greater curvature, medial displacement of the pylorus, and a volvulated ileal loop. The involved bowel appeared congested with areas of early ischemia (Fig. 2).

Derotation and careful assessment of bowel viability were undertaken; due to persistent ischemic changes, resection of the compromised ileal segment with primary mechanical anastomosis was required. The postoperative course was uneventful, and the patient experienced complete resolution of symptoms.

## Discussion

Intestinal malrotation presenting in adulthood is rare, accounting for ~0.2%–0.5% of cases, and represents a diagnostic challenge due to its nonspecific or intermittent symptoms [5]. CT plays a pivotal role in diagnosis, with suggestive findings including abnormal superior mesenteric artery/superior mesenteric vein alignment, malposition of the duodenojejunal junction, and the whirlpool sign [6, 7].

However, this case underscores a key clinical message: preoperative imaging may not accurately predict the location or severity of volvulus, and timely surgical exploration remains mandatory when intestinal ischemia is suspected.

Once malrotation or volvulus is diagnosed or strongly suspected, prompt surgical intervention is required. The Ladd procedure remains the cornerstone of treatment in adults, although bowel resection becomes necessary in the presence of ischemic compromise [3].

Early clinical suspicion and decisive operative management prevented progression to bowel necrosis in this patient. This case emphasizes the importance of considering intestinal malrotation in adults presenting with unexplained bowel obstruction, particularly in the absence of previous abdominal surgery.

## Conclusion

Congenital intestinal malrotation is a rare but important differential diagnosis in adult patients presenting with acute intestinal obstruction. Although CT imaging is essential for evaluation, intraoperative findings ultimately guide surgical management. Early surgical intervention is crucial when intestinal ischemia is suspected, even when preoperative imaging findings are partially misleading. Heightened clinical awareness may improve patient outcomes.
